# Diversity of root system architecture and root-shoot biomass allocation in industrial hemp (*Cannabis sativa* L.)

**DOI:** 10.1371/journal.pone.0339929

**Published:** 2026-02-06

**Authors:** Elisa Y. Morales, Marcus Griffiths, Sumeet P. Mankar, George C. Bagnall, Tyler G. Dowd, Richard Fletcher, John K. McKay, Christopher N. Topp

**Affiliations:** 1 Donald Danforth Plant Science Center, St. Louis, Missouri, United States of America; 2 New West Genetics, Windsor, Colorado, United States of America; 3 Michigan State University, East Lansing, Michigan, United States of America; Louisiana State University College of Agriculture, UNITED STATES OF AMERICA

## Abstract

Roots are major contributors to nutrient acquisition, water absorption, and plant anchoring and stability. However, little is known about the root system of industrial hemp (*Cannabis sativa* L.), an increasingly important crop worth $16 billion annually. Hemp is commonly cultivated for grain as an oilseed, stalk biomass for fiber and industrial materials, but has also had growing interest for its carbon sequestration potential due to its reported deep rooting profile. The objectives of this research were to (1) phenotype a panel of 46 industrially-relevant hemp genotypes, (2) quantify the phenotypic differences of shoot and root traits through 2D image analysis, (3) and to investigate genotype grouping strategies and gene targets that could be useful for crop improvement. To phenotype the root system architecture of multiple hemp genotypes representative of production hemp, a large format raised-bed was developed in a greenhouse in which hemp was planted in rows. Root and shoot traits varied across genotypes, with a difference of 175% in total root length between the largest and smallest genotype, and heritability values ranging from 0.51 to 0.88 for key root traits. A strong positive correlation was found between root and shoot biomass (R = 0.93) suggests coordinated resource allocation strategies across genotypes. Of the 46 genotypes studied, two genotypes consistently showed the greatest differences across most of the traits analyzed in the panel. A root-to-shoot quadrant framework was applied to classify hemp ideotypes based on biomass allocation and architectural traits. In addition, comparative genomic analysis identified 74 candidate root architecture genes in hemp that are orthologous to known regulators in maize, rice, and Arabidopsis. These findings highlight substantial phenotypic diversity in hemp root systems and provide a foundation for developing genotype grouping strategies and selecting breeding targets for mapping populations.

## 1. Introduction

Industrial hemp (*Cannabis sativa* L.) is a versatile row crop cultivated as an oilseed, as well as for fiber and biomass. The value of these products on the global market was estimated at $US7.9B in 2024 with a forecast to grow to over $US26B by 2029 [1]. Hemp has been shown to be effective for phytoremediation and ongoing research is exploring its potential as an energy crop, for its carbon sequestration potential [2], for medicinal and oil products [3], animal feed ingredient [4], paper production [5], and as a sustainable building material [6,7], among many other potential uses [5,8].

Hemp breeding and cultivation was prohibited in the United States until the passage of the 2018 farm bill [9,10]. Due to a long period of prohibition, hemp has not received the same level of plant breeding or genetic selection as more common row crops such as corn or soybean. This presents an opportunity to balance phenological and genetic selection of above-ground with below-ground traits, an aspect often overlooked. Additionally, the review by [11] indicates that hemp as a cultivar, has the potential to provide greater positive economic and environmental impacts with selection of desirable root traits. Modification of root and shoot traits promises to enhance both the crop cash value and its environmental impact.

Roots provide key functions for the plant including resource capture and structural anchorage in the soil. Despite the importance of root development on plant performance, phenotyping of root systems remains challenging [12]. Root system architecture, which refers to the spatial distribution of roots, plays a crucial role in plant anchorage, water and nutrient uptake, and toxicities uptake in the case of phytoremediation [13–15]. Plant roots are the primary means of carbon transfer to the soil [16]. Breeding and deploying crops with extensive root systems through trait-based approaches could greatly enhance environmental sustainability. Breeding with a focus on root traits could also have a positive impact on abiotic stress tolerance [17], yield [18], and ecosystem services such as erosion control [19]. The majority of research on hemp to date has focused on the production chain and end products as listed above, agronomic traits such as biomass yield, grain yield and oil content [20–24], or on crop management methods [25–27]. Only a few studies have characterized roots in hemp, and those primarily focused on the influence of growing conditions on roots, or on the potential of hemp roots as a source of chemical compounds [28–34]. Hemp root systems are reported to grow as deep as 2 m with variation in rooting depth likely among genotypes [28].

In this study, we investigated a panel of 46 hemp genotypes grown in a large format raised-bed system under greenhouse conditions. Using an image-based phenotyping approach, root and shoot traits of eight-week-old plants were characterized to evaluate phenotypic diversity, trait correlations, and heritability. The resulting data was used to develop a foundation for classifying hemp ideotypes and identifying orthologous root genes for further study.

## 2. Materials and methods

### 2.1 Plant materials

Seeds from 46 diverse industrial hemp genotypes were provided by New West Genetics, Inc. (NWG). These include European dual-purpose varieties, Canadian oilseed varieties, Chinese fiber varieties, US high-CBD (cannabidiol) varieties and breeding populations created by NWG derived from crosses among them (S1 Table).

### 2.2 Raised-bed construction and preparation

The experiment was conducted in a large raised-bed structure constructed inside a greenhouse at the Donald Danforth Plant Science Center (38.6745° N, 90.3971° W). The unit measured 6 m × 3 m × 1 m (length × width × height), resulting in an 18 m^3^ growth volume, and was built using dimensional treated lumber. The walls consisted of 5 cm × 30 cm × 304 cm boards (nominal size: 2 in × 12 in × 10 ft) attached horizontally to vertical 5 cm × 10 cm boards (nominal size: 2 in × 4 in) at each end using construction-grade lag screws. A 5 cm × 10 cm × 3 m (nominal size: 2 in × 4 in × 10 ft) cross brace was placed across the bottom to connect the two long sides at the midpoint, and 5 cm × 30.5 cm × 3 m (nominal size: 2 in × 12 in × 10 ft) boards were installed diagonally across each 3 m wall to increase rigidity. At the midpoint of each long wall, a 10 cm × 10 cm vertical post (nominal size: 4 in × 4 in) was positioned externally for additional structural support. A 5 cm × 15 cm × 3 m (nominal size: 2 in × 6 in × 10 ft) board was mounted across the top edges to prevent the walls from spreading as the bed was filled with growth media. To reinforce the structure, a polyester tension band (rated to 40,000 N) was wrapped around the perimeter to maintain the structural integrity under load. Steel angle iron caps were placed at each corner to protect the band from abrasion and to allow tightening without fraying. This design ensured long-term stability of the raised-bed unit under the weight of the growth media.

### 2.3. Growth conditions and experiment design

Plants were grown in a greenhouse between January 5, 2022 and March 8, 2022. Seeds of 46 genotypes were sown in 36-well plug trays containing soil and peat planting medium (50% Jolly Gardener/ 50% peat). Germination rates for all genotypes used were above 70% with 15 evenly germinated plants used for the experiment. The seed source was unfeminized seeds with the males thinned later as detailed below [35]. The trays were kept in a greenhouse with a light sensor connected supplemental lighting set to a day length of 12 hours, temperature 30–35°C both day and night, and a humidity of 50–70% for 16 days. After 16 days of growth, the seedlings were transplanted in a randomized block design with three replicate blocks in a large format grow bed containing turface. Five seedlings per genotype per block were spaced 11.5 cm apart with 20 cm row spacing with an equivalent planting density of low-density grain production (43.5 pl m^−2^). Each block served as an independent replicate containing all genotypes arranged in randomized order. Irrigation was applied using 450 drip stakes (0.5 gallon h^−1^ each), providing a total delivery rate of approximately 47 L m^-2^ h^-1^ across the raised bed. A 15-16-17 fertilizer (Jack’s Professional Water-soluble fertilizer, J.R. Peters Inc., Allentown, PA) at 200 ppm Nitrogen was applied in 15-minute pulses every 45 minutes, three days per week (Monday, Wednesday, and Friday). Tempered water was applied on the remaining four days per week at the same drip rate (Tuesday, Thursday, Saturday, and Sunday). With the drip irrigation and rate used there were no signs of drought or nutrient stress.

The sex of the hemp plants was determined after flowering by visually inspecting each plant and identifying female or male phenotypic markers. Female inflorescences were identified by their abundance of stigma in multiple colas or floral clusters, while male hemp plants did not have stigma protruding from the flower clusters and had pollen sacks. In this study, all identified male plants were removed and discarded, with an average culling of two plants per genotype and block, while female and monoecious plants were kept through to the harvest.

### 2.3. Sample collection and harvest measures

The root and shoot harvest were conducted seven-weeks after transplanting. All genotypes had already flowered at seven-weeks after transplanting and were harvested on the same day. All irrigation was stopped two days before the shoot harvest to allow for the turface to partially dry. The stems of all female plants were tagged with a wrap tag and cut 8 cm above the turface line. The shoots were imaged within one hour of harvest using a Sony ILCE-7 camera equipped with a Sony FE 28 mm f/2.0 lens and a ring light (LS Photography PPH67 ring light) for illumination. A single image of the whole intact shoot was taken per plant on a black background with a poker chip in view for scale at a 1.8 m distance from the lens. The shoot images were analyzed using the program RhizoVision Explorer [36]. Images were converted to grayscale and threshold applied to separate shoot from the background, then the shoots were segmented and skeletonized. After imaging, the samples were dried at 50°C for five days and then weighed to obtain their dry biomass weights (Controlled Environment Room, Darwin Chambers, MO, USA). Eleven imaged-based shoot traits were extracted, as well as shoot biomass and root to shoot mass fraction measures.

For the root harvest, three panels on the side of raised bed were removed for direct access to the roots. Carefully utilizing a vacuum while holding the plant stem, the turface was removed to reveal the roots, minimizing any mechanical damage. Once excavated, the harvested roots were wrapped in wet germination paper before same-day imaging. A single image of the whole root system was taken per plant on a black background with a poker chip in view for scale at a 90 cm distance from the lens. After image capture, images were processed using the whole-root mode in RhizoVision Explorer software to extract measures of root system shape. The images of the intact root were converted to grayscale and a threshold applied to separate the root from the background, then the roots were segmented and skeletonized to obtain 13 whole root image traits [37]. The roots were then cut, spread out on a water-filled clear Perspex tray, and imaged on a flatbed scanner with a transparency unit at 600 dpi (Epson Expression 12000XL, Epson America Inc., CA, USA) to obtain root images suitable for root length extraction. These flatbed scanner root images were analyzed using the broken-root mode in RhizoVision Explorer to extract root length related traits by root class, resulting in a further 23 broken root image traits [36]. After flatbed scanner imaging, the roots were transferred into paper bags and dried at 50°C (Fischer Scientific Isotemp Incubator, PA, USA) for up to 5 days to obtain dry biomass weights, specific root length, and root to shoot mass fraction measures. Root and shoot images and RhizoVision Explorer configuration files are accessible at 10.5281/zenodo.17783092.

### 2.4. *In silico* analysis

The construction of a phylogenetic tree for hemp root genes and the identification of core root system architecture genes was based on previously published reports on root system architecture genes. The primary transcript protein sequences of maize, rice, and *Arabidopsis* root system architecture genes predicted by [38] were downloaded from the Phytozome database (https://data.jgi.doe.gov/). The protein sequences of *Cannabis sativa* (GenBank assembly - GCA_900626175.2) were used for BLAST against the (B73 RefGen v4), rice (MSU RGAP Release 7), and *Arabidopsis* (Araport11) databases using the blastp command. The primary goal was to identify high-confidence, functionally plausible candidate rather than a complete orthogroup assessment. The BLAST results were therefore filtered for a sequence similarity of 95% to prioritize high-confidence orthologs with a stringent identity cutoff to reduce false positives. These results were then combined into a single FASTA file for further analysis. A FASTA result file was used to align the protein sequence in MEGA11 using the MUSCLE option in the MEGA11 GUI [39]. Phylogenetic analysis was conducted using the Maximum Likelihood method with 1000 bootstrap replications. To visualize the orthologous genes among *Cannabis sativa*, *Zea mays*, *Arabidopsis thaliana*, and *Oryza sativa*, a phylogenetic tree was created using FigTree v1.4.4. [40]. BLAST, alignment, and phylogenetic project files for the *in silico* analysis are accessible at 10.5281/zenodo.17870483.

### 2.5. Statistical analysis

Statistical analyses were conducted using R version 4.4.3 [41]. The raw data is available in S1 Data. The following measurements were derived from image analysis and biomass data: Specific Root Length (m^-1^ g^-1^) was calculated as total root length divided by root dry biomass. Root Mass Fraction was calculated as root dry biomass divided by total dry biomass. Lateral Root Length (mm) was determined as the sum of root length within diameter ranges 2 and 3. Lateral Root Fraction was calculated as the proportion of lateral roots to total root length.

The tidyverse packages including dplyr and broom were used for data processing, and ggplot2 and patchwork were used for visualization [42]. Analysis of variance (ANOVA) was conducted using linear mixed-effects models fitted with lme4::lmer(), with genotype as a fixed effect and block as a random effect. ANOVA tables for testing the effect of genotype were obtained from the fitted models using the anova() function. The agricolae package was used to perform post-hoc multiple comparison tests [43]. Broad-sense heritability was using the equation ${H^{\text{2}}=\sigma }_{g}^{\text{2}}/(\sigma_{g}^{\text{2}}+\sigma_{e}^{\text{2}}/\text{r})$. The variables $\sigma_{g}^{\text{2}}$, ${<\sigma }_{e}^{\text{2}}$, and r represent the variances of the genotypic effect, environmental effect, and the number of replicates blocks, respectively. Correlations among the root and shoot traits extracted from the 46 hemp genotypes were explored. Pairwise pearson correlation coefficients were computed using the stats::cor() function with pairwise complete observations to handle missing data. Corresponding significance p-values were calculated using the ggcorrplot::cor_pmat() function [44].

To classify genotypes based on coordinated root and shoot development, percentile ranks were calculated for six key traits: root dry biomass, total root length, root crown convex area, shoot dry biomass, shoot height, and shoot convex area. Percentile ranks were computed using the dplyr::ntile() function, dividing each trait distribution into 100 quantiles. Genotypes were then assigned to one of four quadrants representing major root–shoot allometric patterns using case dplyr::case_when() function. Trait data were mean-centered and scaled prior for visualization and follow up analysis. Principal component analysis (PCA) with K-means clustering was performed using the FactoMineR and factoextra packages to identify natural groupings among genotypes [45]. Bootstrap Jaccard similarity values were calculated using fpc::clusterboot(). Clustering was tested across three and four cluster solutions, and the resulting group assignments were compared to predefined quadrant-based groupings to evaluate concordance between unsupervised clustering and trait-based classifications.

## 3. Results

### 3.1. Phenotypic variation in hemp genotypes for root and shoot traits

In a raised-bed growth system, 46 hemp genotypes were grown and harvested after seven weeks. Individual root and shoot system photos were taken for each plant, resulting in 637 images, and an additional 375 flatbed scanner images were taken to accurately determine root length. Root and shoot phenotypes were extracted from the images, and significant phenotypic differences were observed for multiple root and shoot traits (S2 Table).

**Fig 1:**
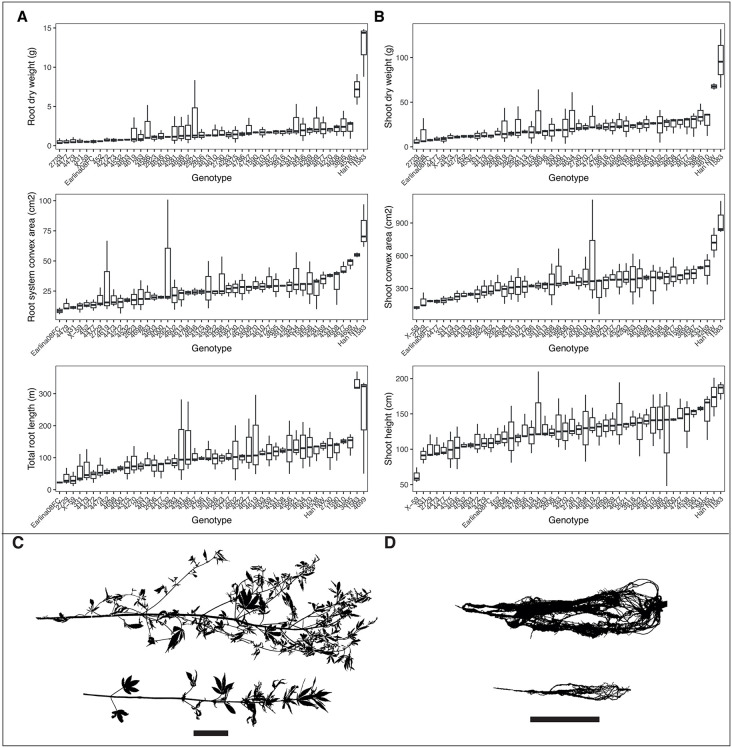
Large diversity observed in root and shoot size traits among 46 hemp genotypes. **(A)** Boxplots for the following extracted root traits. Root dry biomass, convex hull area, and total root length. **(B)** Boxplots for the following extracted shoot traits. Shoot dry weight, convex hull area, and height. **(C, D)** Representative greyscale root and shoot images for a small and large genotype, genotype 2729 and genotype 1583, respectively. Scale bar = 20 cm. Data from S3 Table.

For root length traits, significant genotypic differences were observed including total root length, lateral and second-order lateral root lengths, average root diameter, and specific root length (p < 0.05, S2 Table). Heritable variation for root length and biomass traits was found in hemp with broad-sense heritability (H^2^) scores of 0.63 for total root length, 0.5 for average root diameter, and 0.47 for specific root length (S3 Table). Broad-sense heritability represents the proportion of phenotypic variance explained by genetic differences under the specific environment tested in the study; values above 0.4 indicate a moderate to high genetic contribution. Genotype 1583 had the greatest mean root length, which was 175% larger than the smallest genotype, Earlina08FC (Fig 1A, S4 Table). For root system convex area, genotype 1583 had the greatest area which was 160% larger than the smallest genotype Earlina08FC (Fig 1A, S4 Table). Genotype 1583 and Earlina08FC also had the largest and smallest lateral root fractions, with Genotype 1583 having a 42% greater fraction than Earlina08FC (S4 Table). For specific root length, genotype 4603 had the greatest value at 153 m g^-1^, whereas genotype Han NW had the smallest specific root length at 20 mg^-1^ (S4 Table).

For the root system shape and distribution traits, significant genotypic differences were observed including for root convex network area, root crown maximum width, and lower root area (S2 Table). Root system shape and distribution traits were found to be heritable with broad-sense heritability scores of 0.64 for root convex area, 0.51 for lower root area, and 0.45 for root crown maximum width (S3 Table). Genotype 1583 had the largest convex hull area which was 160% larger than the smallest genotype Earlina08FC (Fig 1A). Genotype 1583 also had the largest root crown maximum width, which was 105% wider compared to the smallest width of genotype 4479 (S4 Table). For lower root area, genotype 1583 had the largest area with a 172% increased area compared to the smallest genotype 4603 (S4 Table).

For the measured shoot traits, large differences among the 46 hemp genotypes were observed (Fig 1B). Significant genotypic differences were observed for shoot size and distribution traits, including biomass, height, network area, and ratio of shoot width to height (p < 0.05, S2 Table). Shoot traits were highly heritable with broad-sense heritability scores of 0.77 for shoot dry biomass, 0.66 for shoot convex area, and 0.50 for shoot height (S3 Table). Genotype 2729 exhibited the smallest shoot biomass, while genotype 1583 had the largest shoot biomass with a 175% increase (Fig 1B). Genotype X-59 had the smallest shoot convex hull area, whereas genotype 1583 again was the largest with a 153% greater convex hull area (Fig 1B, S4 Table). For shoot distribution traits, genotype 4000 had the smallest width-to-depth ratio at 0.24, while genotype X-59 had the largest ratio, which was 71% greater at 0.54 (S4 Table). In addition, there was a significant genotypic effect for root to shoot mass fraction with genotype 4479 having the smallest mass fraction of 0.045 and genotype 4646 with the greatest root to shoot mass fraction of 0.14 (p < 0.05, S3 Table, S4 Table).

Post-culling counts by genotype and block were used to conduct a limited sensitivity check excluding plants from plots with the most extreme culling (S2 Data). Approximately 12% of plots showed substantial culling (≥4 removed plants) and were excluded from this analysis. The primary genotype rankings, heritability estimates, and major root–shoot correlations were unaffected. Three traits were no longer significant for genotypic effects once heavily culled plots were excluded (root mass fraction, root lateral fraction, and root median diameter, ns). Conversely, two traits that were previously non-significant became significant (root crown average hole size and shoot lower area; p < 0.05).

### 3.2. Correlation among traits

**Fig 2:**
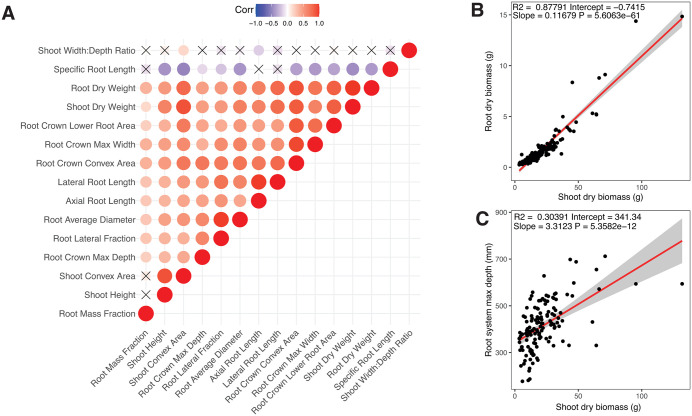
Phenotype correlations among 46 hemp genotypes. **(A)** Correlation matrix with positive correlations between traits depicted in red color, negative correlations in blue color, and X symbol marks all bivariate correlations that were not significant with a P value set at 0.05. **(B)** Linear regression between shoot dry biomass and root dry biomass. **(C)** Regression between shoot dry biomass and root system maximum depth. Data from S5 Table.

Analysis of the whole dataset revealed a major cluster of positively correlated traits encompassing biomass traits, plant size traits, and root and shoot shape traits (Fig 2A, S5 Table). In contrast, the majority of plant traits were negatively associated with specific root length. For shoot width to depth ratio, little to no correlation with other plant traits was observed. A strong positive correlation was observed between root and shoot biomass (R = 0.93, p < 0.01, Fig 2B), and a similar moderate relationship was found between shoot biomass and root system maximum depth (R = 0.50, p < 0.01, Fig 2C).

### 3.3. Hemp genotype quadrant grouping by root and shoot size traits

**Fig 3:**
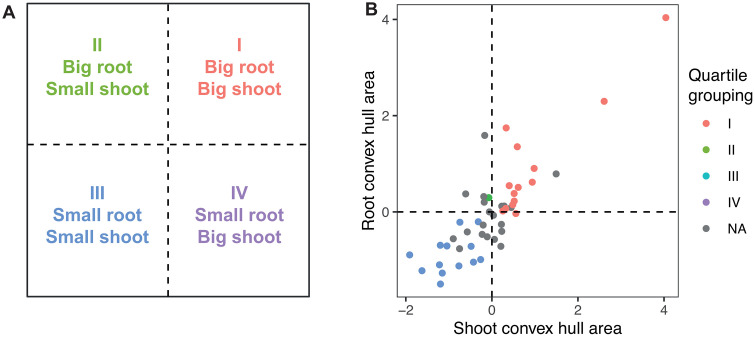
Hemp genotype quadrant grouping by root and shoot size traits. **(A)** Quadrant designations assigned based on 50th percentiles for multiple root and shoot size traits (Root biomass, total root length, root convex hull area, shoot biomass, shoot convex hull area, shoot height). **(B)** Normalized root and shoot convex hull data with the multiple trait quadrant color assignment. If a genotype did not fulfill the requirements of 50th percentiles for each trait then they were grouped as NA.

Hemp genotypes were classified into four groupings by root and shoot size traits (S6 Table). Four groupings were devised: (I) big root and big shoot, (II) big root and small shoot, (III) small root and small shoot, (IV) small root and big shoot (Fig 3A). The phenotypes used for the classification were root biomass, total root length, root convex area, shoot biomass, and shoot convex area. Percentile rankings were assigned to each hemp genotype per phenotype and the phenotype data scaled. Quadrant groupings were assigned when a genotype fulfilled the upper or lower 50% percentile of each respective phenotype (Fig 3B). The majority of the hemp genotypes fell into group I and group III, with 16 and 15 genotypes, respectively. One genotype was assigned into group II and one genotype was assigned to group IV. The remaining 13 genotypes did not fulfil all requirements to be assigned a quadrant group, one genotype resembled closely to group I, four genotypes resembled group II, five genotypes resembled group III, and three genotypes to group IV. K-means clustering of the PCA results largely supported the initial genotype groupings (S1 Fig). When clustered into three groups, most genotypes in group I and group III corresponded to clusters 1 and 3, respectively (S6 Table). Increasing the number of clusters to four introduced more fine-scale genotype differences, however groups I and III remained in general distinct, and the genotypes that had not fulfilled a quadrant grouping were also in a separate and distinct cluster (S6 Table). Bootstrap Jaccard similarity values indicated low–moderate stability for the three-cluster solution (0.43–0.52). With four-clusters it showed one highly stable cluster (0.81) and three clusters of moderate stability (0.43–0.60).

### 3.4. Identification of root candidate genes in Cannabis in hemp

**Fig 4:**
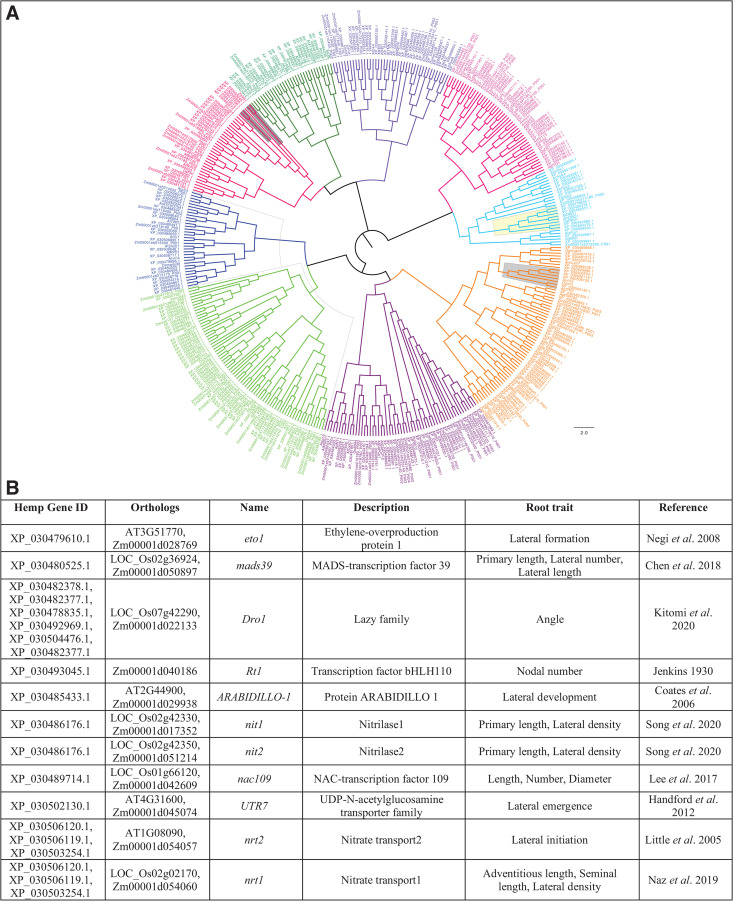
Dissection of priority root candidate genes for hemp. **(A)** Maximum likelihood phylogenetic tree of 301 *Cannabis sativa* genes identified as orthologs of known RSA regulators from *Arabidopsis thaliana*, *Oryza sativa* (rice), and *Zea mays* (maize). Protein sequences were aligned using MUSCLE, the tree was constructed in MEGA11 with 1,000 bootstrap replicates, visualized in FigTree. Gene clusters were grouped based on ≥95% sequence similarity and are color-coded into nine distinct functional clusters. **(B)** Key root architecture genes identified through phylogenetic analysis, along with their known functions and representative homologs from model species. (For additional genes, refer to S7 Table).

Despite the growing economic and ecological interest in *Cannabis sativa*, little is known about the genetic regulation of its root traits due to a historical lack of genetic resources and breeding efforts. To address this, we have employed root phenotyping and *in silico* comparative genomics to provide a framework for dissecting root system architecture (RSA) in industrial hemp. The phenotyping effort revealed substantial and heritable variation in root traits such as total root length, convex hull area, and biomass among 46 diverse hemp genotypes, highlighting the potential for genetic improvement. Linking this phenotypic diversity with underlying genetic determinants, an *in silico* analysis identified a total of 301 root system architecture-related orthologues from maize, rice, and *Arabidopsis*, including 74 high-confidence hemp candidate genes with functional relevance (S7 Table). Duplicate gene entries represent genes that are orthologous to multiple loci in maize, rice, or *Arabidopsis*. These RSA genes were primarily grouped into four clusters and further divided into nine clusters based on phylogenetic analysis using a 95% sequence similarity threshold for these genes (Fig 4). Among these 301 genes, 74 root genes identified as homologs of known RSA genes which have potential control of root system architecture in hemp (S7 Table). The detailed list of homologous hemp genes with known root system architecture genes from maize, rice, and Arabidopsis is provided in S7 Table.

Key root genes were primarily highlighted in grey (7 genes), yellow (8 genes), and dark grey (5 genes). Among them, seven genes that have been functionally characterized in maize (*ZmDRO1*, *ZmRt1*), rice (*OsNRT1*, *OsDRO1*), and Arabidopsis (*AtDro1*, *AtNRT2*.1, *AtUTR7*) were identified. The *XP_030478835.1, XP_030482378.1, XP_030490379.1,* and *XP_030490380* genes are homologs of *DEEPER ROOTING 1* (*DRO1*), an auxin-response gene that is directly regulated by Auxin Response Factors that control root growth angle and promote deep rooting [17]. The maize *rootless1 (ZmRt1)* encodes a *bHLH* transcription factor that governs nodal root development in maize and shares phylogenetic similarity with three hemp genes (*XP_030484730.1*, *XP_030481898.1,* and *XP_030484738.1*)*.* Seven genes homologous to the Arabidopsis and rice genes (*AtNRT2.1* and *OsNRT1*), known nitrogen transporters, were identified, which might be potentially responsible for the nitrogen transport pathway in hemp [46,47]. A homolog (XP_030502130.1) to AT4G31600 (*AtUTR7*) encodes a Golgi-localized UDP–glucose/UDP–galactose transporter that regulates lateral root emergence in Arabidopsis was also identified [48]. This genome-wide analysis underscores the conservation of RSA regulatory networks across plant species and provides a curated gene set for downstream validation in hemp. These genes are prime candidates for genetic manipulation via marker-assisted selection or gene-editing technologies to improve RSA-related traits in breeding programs. A stringent identity cutoff was employed to reduce false positives and to focus on a conservative set of orthologs with the strongest likelihood of functional equivalence and therefore this approach prioritizes confidence over completeness and may under-represent the total number of true functional homologs. Further development of breeding populations and validation with experimental evidence will be necessary to confirm the functions of candidate genes related to root traits.

## 4. Discussion

Industrial hemp has very limited studies focusing on its root traits despite its economic value and potential as a sustainable crop. The purpose of this study was to address this knowledge gap by providing a comprehensive phenotypic analysis of root and shoot traits of 46 diverse hemp genotypes. Crop improvement programs have historically prioritized above-ground traits such as yield and flowering time, while below-ground traits have received comparatively little attention. This oversight has persisted despite growing evidence that root system architecture plays a critical role in nutrient uptake, water use efficiency, anchorage, and resilience under abiotic stress [17,49].

Evaluation of root traits has been constrained by logistical and methodological challenges. However, in recent years, advances in image-based phenotyping and increased access to such approaches have significantly reduced these barriers to root research [36,50,51]. In this study, substantial variation was observed among hemp genotypes for both root and shoot traits, including total root length, root convex area, and biomass, with several traits exhibiting moderate to high broad-sense heritability. For example, heritability for root length was 0.63 and for shoot biomass was 0.78, suggesting that both traits exhibit substantial genetic variance and could respond to selection. These estimates reflect genetic contributions to trait variation within the context of this greenhouse environment. Genotype 1583 consistently exhibited large root and shoot traits, whereas genotype 4479 represented a more compact phenotype, suggesting potential contrasting ideotypes. Importantly, we found a strong positive correlation between shoot and root biomass, aligning with studies in other crops where shoot vigor and root capacity often co-vary [52–55]. However, specific traits, such as specific root length, were negatively correlated with biomass, indicating trade-offs [56,57].

Hemp is known as dioecious, meaning that the male and female reproductive organs are found in separate individuals, but occasionally, monoecious male plants will develop before female flowers in less defined clusters in the upper nodes of the stem [58]. In this study, the female and monoecious plants were evaluated, and the male plants were removed upon identification during growth. Differences in males and females are reflective of overall patterns of sexual dimorphism, where females tend to be larger than males in dioecious species [59,60]. In hemp specifically, female plants have been shown to produce 43% more aboveground biomass than monoecious cultivars and 63% more than males, and the root systems of female plants were on average two times larger than those of males [33]. We observed substantial variation in root and shoot traits in this study, which included only female and monoecious plants present.

Our root-to-shoot quadrant analysis revealed four putative groupings of hemp ideotypes. Most genotypes clustered into either the big root-big shoot or small root-small shoot categories, reflecting common allometric scaling. Only one genotype was classified as big root-small shoot, and one as small root-big shoot, though several genotypes partially matched these ideotypes. These groupings provide a practical framework for breeders aiming to match root architecture with specific production goals such as drought tolerance (deep rooting), nutrient uptake efficiency (dense fine roots), or biomass allocation (greater shoot productivity) [56,61,62]. Expanding the germplasm panel to include a wider range of genotypes and evaluating them under field-conditions and controlled environments with varied photoperiods would likely reveal additional variation in both trait expression and quadrant classification. Incorporating additional root phenotypes, such as root length density by soil depth, root growth rate, or root hair density, could further refine this ideotype framework and enhance its utility for breeding programs [63–65].

It is important to note that these results were obtained under controlled greenhouse conditions with a 12-hour photoperiod, temperatures of 30–35 °C, and a planting density typical of low-density grain production. This regime likely constrained the vegetative and root potential of late-flowering, photoperiod-sensitive genotypes while favoring more day-neutral or short-day–adapted lines. Industrial hemp varieties have recently been classified by discrete photoperiod-sensitivity with influence to vegetative and reproductive allocation under different daylengths [66]. Short days combined with heat accelerate flowering and limit vegetative biomass production in a genotype-dependent manner [67]. Also, the culled male plants would have allowed the neighbouring plants to more space to grow. Culling rate was on average two plants per genotype per block but as stand heterogeneity could influence genotype means, heritability estimates, and root–shoot associations a limited sensitivity check was conducted that excluded highly culled plots. There was no obvious impact of culling rate between the largest and smallest genotypes in this study, with primary genotype rankings, heritability estimates, and major root–shoot correlations were unaffected.

In addition, a soilless substrate was used to facilitate uniform growth conditions and enable accurate recovery and imaging of root systems. While this medium supports reproducible measurements of root length and architecture, it does not fully replicate the mechanical resistance, nutrient gradients, or microbial complexity of field soils [29,68,69]. Therefore, the root-to-shoot quadrant groupings and trait relationships identified here should be interpreted as performers and phenotypic associations under these warm, short-day controlled conditions, rather than as absolute indicators across environments and generalizable genetic allocation patterns.

The *in silico* identification of conserved RSA genes addresses a vital gap in hemp genomics. By leveraging comparative genomics, we demonstrated that hemp shares a conserved root regulatory network with model species, including key genes such as *DRO1*, *Rt1*, *NRT1*, and *UTR7*. These genes are crucial in determining root shape, depth, architecture, and nutrient responsiveness. The identification of 74 high-confidence orthologs provides a valuable foundation for targeted breeding and functional validation efforts. The *DRO1* homologs, for instance, could be used to promote deeper rooting for better drought resistance, while *NRT*-related genes provide ways to improve nitrogen uptake efficiency, and *UTR7* is associated with lateral root emergence.

This dual approach, combining phenotypic characterization with genome-wide gene discovery, improves our ability to establish predictive links between traits and underlying genetic mechanisms. Additionally, the availability of this gene resource can accelerate the development of hemp varieties tailored to different soil environments and resource conditions. Future research should focus on experimentally validating these genes through gene expression analysis, mutagenesis, and field testing. Integrating multi-omics datasets, such as transcriptomics and metabolomics, will also help to clarify gene-trait relationships and reveal regulatory interactions. Ultimately, this work lays a foundational step toward both conventional and molecular breeding strategies that enhance hemp productivity, sustainability, and resilience.

## 5. Conclusion

This study provides a comprehensive assessment of root system architecture in industrial hemp. We demonstrate that hemp exhibits significant and heritable variation in both root and shoot traits and present a framework for classifying ideotypes based on biomass allocation and root distribution. This approach could support modern breeding pipelines aiming to incorporate below-ground and above-ground phenotypes. Additionally, our identification of putative root-related gene homologs provides a foundation for future genetic studies and may ultimately inform targeted breeding efforts aimed at improving productivity, resource efficiency, and carbon sequestration in hemp. These targets are of importance for hemp as a phytoremediation crop, as well as in the building, textile, medical, food, and biofuel industries.

## Supporting information

S1 Fig K-means clustering of trait data for hemp genotypes with (A) 3 groups and (B) 4 groups. (DOCX)

S1 Table Genotype information. (CSV)

S2 Table Statistical analysis output. (CSV)

S3 Table Summary statistics across all genotypes. (XLSX)

S4 Table Mean data across genotyps. (CSV)

S5 Table Correlation data. (XLSX)

S6 Table Percentile ranking of genotypes. (CSV)

S7 Table Key root genes identified in phylogenetic analysis. (XLSX)

S1 Data Raw data. (CSV)

S2 Data Data check with culled genotypes removed. (XLS)

## References

[1] Technavio. Oilseeds market analysis North America, APAC, Europe, South America, Middle East and Africa - US, China, India, Germany, Brazil - size and forecast 2024-2028. 2024.

[2] Finnan J, Styles D. Hemp: A more sustainable annual energy crop for climate and energy policy. Energy Policy. 2013;58:152–62. doi: 10.1016/j.enpol.2013.02.046

[3] Visković J, Zheljazkov VD, Sikora V, Noller J, Latković D, Ocamb CM, et al. Industrial Hemp (Cannabis sativa L.) Agronomy and Utilization: A Review. Agronomy. 2023;13(3):931. doi: 10.3390/agronomy13030931

[4] Kalaitsidis K, Parissi Z, Theodoridis A, Tsaliki E, Vasilopoulou K, Dokou S, et al. Evaluation of hemp cake (Cannabis sativa) and other hemp by-products of Greek origin and efficacy in dairy cow nutrition. Archiva Zootechnica. 2023;26(2):149–70. doi: 10.2478/azibna-2023-0020

[5] Sieracka D, Frankowski J, Wacławek S, Czekała W. Hemp biomass as a raw material for sustainable development. Appl Sci. 2023;13(17):9733.

[6] Arehart JH, Nelson WS, Srubar WV. On the theoretical carbon storage and carbon sequestration potential of hempcrete. J Clean Prod. 2020;266:121846.

[7] Pervaiz M, Sain MM. Carbon storage potential in natural fiber composites. Resour Conserv Recycl. 2003;39(4):325–40.

[8] Ribeiro J, Bueno G, Martín MR, Rocha J. Experimental Study on Mechanical Properties of Hemp Fibers Influenced by Various Parameters. Sustainability. 2023;15(12):9610. doi: 10.3390/su15129610

[9] Conaway KM. HR 2-115th Congress (2017-2018): Agriculture Improvement Act of 2018. 2018. https://www.aaea.org/publications/publications-update/mcmorris-rodgers-statement-on-passage-of-2018-farm-bill

[10] Adesina I, Bhowmik A, Sharma H, Shahbazi A. A review on the current state of knowledge of growing conditions, agronomic soil health practices and utilities of hemp in the United States. Collect FAO Agric. 2020;10(4):129.

[11] Rehman M, Fahad S, Du G, Cheng X, Yang Y, Tang K, et al. Evaluation of hemp (Cannabis sativa L.) as an industrial crop: a review. Environ Sci Pollut Res Int. 2021;28(38):52832–43. doi: 10.1007/s11356-021-16264-5 34476693

[12] Ryan PR, Delhaize E, Watt M, Richardson AE. Plant roots: understanding structure and function in an ocean of complexity. Ann Bot. 2016;118(4).

[13] Comas LH, Becker SR, Cruz VMV, Byrne PF, Dierig DA. Root traits contributing to plant productivity under drought. Front Plant Sci. 2013;4:442. doi: 10.3389/fpls.2013.00442 24204374 PMC3817922

[14] Riedelsberger J, Blatt MR. Editorial: Roots-The Hidden Provider. Front Plant Sci. 2017;8:1021. doi: 10.3389/fpls.2017.01021 28659960 PMC5468936

[15] Morgan JB, Connolly EL. Plant-soil interactions: nutrient uptake. Nature Education Knowledge. 2013;4(8):2.

[16] Kell DB. Breeding crop plants with deep roots: their role in sustainable carbon, nutrient and water sequestration. Ann Bot. 2011;108(3):407–18. doi: 10.1093/aob/mcr175 21813565 PMC3158691

[17] Uga Y, Sugimoto K, Ogawa S, Rane J, Ishitani M, Hara N, et al. Control of root system architecture by DEEPER ROOTING 1 increases rice yield under drought conditions. Nat Genet. 2013;45(9):1097–102. doi: 10.1038/ng.2725 23913002

[18] Narayanan S, Mohan A, Gill KS, Prasad PVV. Variability of root traits in spring wheat germplasm. PLoS One. 2014;9(6):e100317. doi: 10.1371/journal.pone.0100317 24945438 PMC4063797

[19] Ola A, Dodd IC, Quinton JN. Can we manipulate root system architecture to control soil erosion?. Soil. 2015;1(2):603–12.

[20] Struik PC, Amaducci S, Bullard MJ, Stutterheim NC, Venturi G, Cromack HTH. Agronomy of fibre hemp (Cannabis sativa L.) in Europe. Industrial Crops and Products. 2000;11(2–3):107–18. doi: 10.1016/s0926-6690(99)00048-5

[21] Amaducci S, Zatta A, Pelatti F, Venturi G. Influence of agronomic factors on yield and quality of hemp (Cannabis sativa L.) fibre and implication for an innovative production system. Field Crops Research. 2008;107(2):161–9. doi: 10.1016/j.fcr.2008.02.002

[22] Amaducci S, Errani M, Venturi G. Plant Population Effects on Fibre Hemp Morphology and Production. Journal of Industrial Hemp. 2002;7(2):33–60. doi: 10.1300/j237v07n02_04

[23] Frankowski J, Przybylska-Balcerek A, Graczyk M, Niedziela G, Sieracka D, Stuper-Szablewska K. The Effect of Mineral Fertilization on the Content of Bioactive Compounds in Hemp Seeds and Oil. Molecules. 2023;28(12):4870. doi: 10.3390/molecules28124870 37375430 PMC10302684

[24] Serkov VA, Koshelyaev VV, Davydova OK, Koshelyaeva IP. Evaluation Of New Initial Material In Breeding Of Monoecious Industrial Hemp For Seed Productivity And Oil Content. NP. 2024;1(4 (72)). doi: 10.36461/np.2024.72.4.003

[25] van der Werf HMG. Life Cycle Analysis of field production of fibre hemp, the effect of production practices on environmental impacts. Euphytica. 2004;140(1–2):13–23. doi: 10.1007/s10681-004-4750-2

[26] van der Werf HMG, Wijlhuizen M, de Schutter JAA. Plant density and self-thinning affect yield and quality of fibre hemp (Cannabis sativa L.). Field Crops Research. 1995;40(3):153–64. doi: 10.1016/0378-4290(94)00103-j

[27] Amaducci S, Gusovius H. Hemp – Cultivation, Extraction and Processing. Industrial Applications of Natural Fibres. Wiley. 2010. 109–34. 10.1002/9780470660324.ch5

[28] Amaducci S, Zatta A, Raffanini M, Venturi G. Characterisation of hemp (Cannabis sativa L.) roots under different growing conditions. Plant Soil. 2008;313(1):227.

[29] Huang S, Li H, Xu J, Zhou H, Seeram NP, Ma H, et al. Chemical constituents of industrial hemp roots and their anti-inflammatory activities. J Cannabis Res. 2023;5(1):1. doi: 10.1186/s42238-022-00168-3 36642726 PMC9841654

[30] Mortas M, Besir A. Industrial hemp root: Optimum infusion parameters for alternative extracted beverage products. Food Chemistry Advances. 2023;3:100487. doi: 10.1016/j.focha.2023.100487

[31] Kaminsky N, Hubert J, Guerin C, Mazlani M, Kotland A, Pozzobon V, et al. Deciphering the Phytochemical Potential of Hemp Hairy Roots: A Promising Source of Cannabisins and Triterpenes as Bioactive Compounds. Molecules. 2024;29(23):5792. doi: 10.3390/molecules29235792 39683949 PMC11643499

[32] Kim Y, Kim W, Kim S-H, Sim K-S, Kim K-H, Cho K-H, et al. Protective Effects of Hemp (Cannabis sativa) Root Extracts against Insulin-Deficient Diabetes Mellitus In Mice. Molecules. 2023;28(9):3814. doi: 10.3390/molecules28093814 37175224 PMC10179809

[33] McGrail RK, Nelson JA, Pearce RC, McCulley RL. Hemp root system architecture and allometric relationships vary between monoecious and dioecious cultivars. Agrosystems Geosci & Env. 2025;8(2). doi: 10.1002/agg2.70123

[34] Bajwa P, Singh S, Kafle A, Singh M, Saini R, Trostle C. Impact of planting dates and seeding densities on soil water depletion pattern, root distribution, and water productivity of industrial hemp. Farming System. 2025;3(3):100152. doi: 10.1016/j.farsys.2025.100152

[35] Carey SB, Bentz PC, Lovell JT, Akozbek LM, Myers ZA, Korani W, et al. An X-linked sex determination mechanism in cannabis and hop. bioRxiv. 2025;:2024.12.09.627636. doi: 10.1101/2024.12.09.627636 42192122 PMC13389243

[36] Seethepalli A, Dhakal K, Griffiths M, Guo H, Freschet GT, York LM. RhizoVision Explorer: open-source software for root image analysis and measurement standardization. AoB Plants. 2021;13(6):plab056. doi: 10.1093/aobpla/plab056 34804466 PMC8598384

[37] Seethepalli A, Guo H, Liu X, Griffiths M, Almtarfi H, Li Z, et al. RhizoVision Crown: An Integrated Hardware and Software Platform for Root Crown Phenotyping. Plant Phenomics. 2020;2020:3074916. doi: 10.34133/2020/3074916 33313547 PMC7706346

[38] He K, Zhao Z, Ren W, Chen Z, Chen L, Chen F, et al. Mining genes regulating root system architecture in maize based on data integration analysis. Theor Appl Genet. 2023;136(6):127. doi: 10.1007/s00122-023-04376-0 37188973

[39] Tamura K, Stecher G, Kumar S. MEGA11: Molecular Evolutionary Genetics Analysis version 11. Mol Biol Evol. 2021;38(7):3022–7. 33892491 10.1093/molbev/msab120 PMC8233496

[40] Rambaut A. Figtree. https://github.com/rambaut/figtree/releases/tag/v1.4.4 2025 June 9.

[41] R Core Team. R: A Language and Environment for Statistical Computing. Vienna, Austria: R Foundation for Statistical Computing. 2025.

[42] Wickham H, Averick M, Bryan J, Chang W, McGowan L, François R, et al. Welcome to the Tidyverse. JOSS. 2019;4(43):1686. doi: 10.21105/joss.01686

[43] De Mendiburu F. Package “agricolae.”. 2023.

[44] Kassambara A. Package “ggcorrplot.”. 2023.

[45] Husson F, Josse J, Le S, Mazet J. FactoMineR: Multivariate Exploratory Data Analysis and Data Mining. https://cran.r-project.org/web/packages/FactoMineR/FactoMineR.pdf 2025. 2025 October 22.

[46] Zhou J, Wang X, He Y, Sang T, Wang P, Dai S. Identification of a novel nitrate transporter gene in Arabidopsis thaliana (AtNRT2.1) required for nitrate uptake and utilization. Plant Journal. 2002;29(4):415–25.

[47] Fan X, Tang Z, Tan Y, Zhang Y, Luo B, Yang M, et al. Overexpression of a pH-sensitive nitrate transporter in rice increases crop yields. Proc Natl Acad Sci U S A. 2016;113(26). 10.1073/pnas.1525184113 PMC4932942 27274069

[48] Handford M, Rodríguez-Furlán C, Marchant L, Segura MF, Gómez D, Foresti O. Arabidopsis thaliana UDP-galactose/UDP-glucose transporter AtUTr7 is required for lateral root emergence. Journal of Experimental Botany. 2012;63(13):4917–30.

[49] Zhan A, Lynch JP. Reduced frequency of lateral root branching improves N capture from low-N soils in maize. J Exp Bot. 2015;66(7):2055–65. doi: 10.1093/jxb/erv007 25680794 PMC4378636

[50] Smith AG, Han E, Petersen J, Olsen NAF, Giese C, Athmann M, et al. RootPainter: deep learning segmentation of biological images with corrective annotation. New Phytol. 2022;236(2):774–91. doi: 10.1111/nph.18387 35851958 PMC9804377

[51] Zeng D, Li M, Jiang N, Ju Y, Schreiber H, Chambers E, et al. TopoRoot: a method for computing hierarchy and fine-grained traits of maize roots from 3D imaging. Plant Methods. 2021;17(1):127. doi: 10.1186/s13007-021-00829-z 34903248 PMC8667396

[52] Bai C, Liang Y, Hawkesford MJ. Identification of QTLs associated with seedling root traits and their correlation with plant height in wheat. J Exp Bot. 2013;64(6):1745–53. doi: 10.1093/jxb/ert041 23564959 PMC3617839

[53] Demissie HS, Mindaye TT, Teklu DN, Kebede FG. Root system architecture analysis of sorghum genotypes and its effect on drought adaptation. Rhizosphere. 2023;27:100772. doi: 10.1016/j.rhisph.2023.100772

[54] Louvieaux J, Spanoghe M, Hermans C. Root morphological traits of seedlings are predictors of seed yield and quality in winter oilseed rape hybrid cultivars. Front Plant Sci. 2020;11:568009. doi: 10.3389/fpls.2020.568009 33178235 PMC7593254

[55] Yang JC, Zhang H, Zhang JH. Root morphology and physiology in relation to the yield formation of rice. J Integr Agric. 2012;11(6).

[56] Chen W, Wu Y, Fritschi FB, Juenger TE. The genetic basis of the root economics spectrum in a perennial grass. Proc Natl Acad Sci U S A. 2021;118(47):e2107541118. 10.1073/pnas.2107541118 PMC8617459 34799444

[57] Freschet GT, Swart EM, Cornelissen JHC. Integrated plant phenotypic responses to contrasting above- and below-ground resources: key roles of specific leaf area and root mass fraction. New Phytol. 2015;206(4):1247–60. doi: 10.1111/nph.13352 25783781

[58] Hall J, Bhattarai SP, Midmore DJ. Review of Flowering Control in Industrial Hemp. Journal of Natural Fibers. 2012;9(1):23–36. doi: 10.1080/15440478.2012.651848

[59] Barrett SCH, Hough J. Sexual dimorphism in flowering plants. J Exp Bot. 2013;64(1):67–82. doi: 10.1093/jxb/ers308 23183260

[60] Petit J, Salentijn EMJ, Paulo M-J, Thouminot C, van Dinter BJ, Magagnini G. Genetic variability of morphological, flowering, and biomass quality traits in hemp (Cannabis sativa L.). Front Plant Sci. 2020;11:102. 32153610 10.3389/fpls.2020.00102 PMC7044243

[61] Krouk G, Lacombe B, Bielach A, Perrine-Walker F, Malinska K, Mounier E, et al. Nitrate-regulated auxin transport by NRT1.1 defines a mechanism for nutrient sensing in plants. Dev Cell. 2010;18(6):927–37. doi: 10.1016/j.devcel.2010.05.008 20627075

[62] Gao Y, Lynch JP. Reduced crown root number improves water acquisition under water deficit stress in maize (Zea mays L.). J Exp Bot. 2016;67(15):4545–57. doi: 10.1093/jxb/erw243 27401910 PMC4973737

[63] York LM. Functional phenomics: an emerging field integrating high-throughput phenotyping, physiology, and bioinformatics. J Exp Bot. 2019;70(2):379–86. doi: 10.1093/jxb/ery379 30380107

[64] Kuppe CW, Kirk GJD, Wissuwa M, Postma JA. Rice increases phosphorus uptake in strongly sorbing soils by intra-root facilitation. Plant Cell Environ. 2022;45(3):884–99. doi: 10.1111/pce.14285 35137976

[65] Lynch JP, Mooney SJ, Strock CF, Schneider HM. Future roots for future soils. Plant Cell Environ. 2022;45(3):620–36. doi: 10.1111/pce.14213 34725839 PMC9299599

[66] Ansari O, De Prato L, Slaski J. A photoperiod-based classification of industrial hemp (Cannabis sativa L.) and its agronomic implications. Industrial Crops and Products. 2025;233:121431. doi: 10.1016/j.indcrop.2025.121431

[67] De Prato L, Ansari O, Hardy GEStJ, Howieson J, O’Hara G, Ruthrof KX. The cannabinoid profile and growth of hemp (Cannabis sativa L.) is influenced by tropical daylengths and temperatures, genotype and nitrogen nutrition. Industrial Crops and Products. 2022;178:114605. doi: 10.1016/j.indcrop.2022.114605

[68] Mai TH, Schnepf A, Vereecken H, Vanderborght J. Continuum multiscale model of root water and nutrient uptake from soil with explicit consideration of the 3D root architecture and the rhizosphere gradients. Plant Soil. 2018;439(1–2):273–92. doi: 10.1007/s11104-018-3890-4

[69] Schmidt JE, Bowles TM, Gaudin ACM. Using ancient traits to convert soil health into crop yield: impact of selection on maize root and rhizosphere function. Frontiers in Plant Science. 2016;7:373. doi: 10.3389/fpls.2016.00373 27066028 PMC4811947

