# Correction: Diversity of root system architecture and root-shoot biomass allocation in industrial hemp (Cannabis sativa L.)

**DOI:** 10.1371/journal.pone.0349068

**Published:** 2026-05-07

**Authors:** The PLOS One Staff

## Notice of Republication

This article was republished on April 13, 2026, to correct an error in the author list: the author Tyler Dowd was omitted in error. The publisher apologizes for the error. Please download this article again to view the correct version. The originally published, uncorrected article and the republished, corrected articles are provided here for reference.

## Supporting information

S1 File Originally published, uncorrected article. (PDF)

S2 File Republished, corrected article. (PDF)
