# Supplementary figures and images for: Diversity of root system architecture and root-shoot biomass allocation in industrial hemp (Cannabis sativa L.)
Source: PLoS One. 2026 Feb 6;21(2):e0339929. doi: 10.1371/journal.pone.0339929 (PMC12880667; doi:10.1371/journal.pone.0339929)

**Figure S1. K-means clustering of trait data for hemp genotypes with (A) 3 groups and (B) 4 groups.**

**A B**


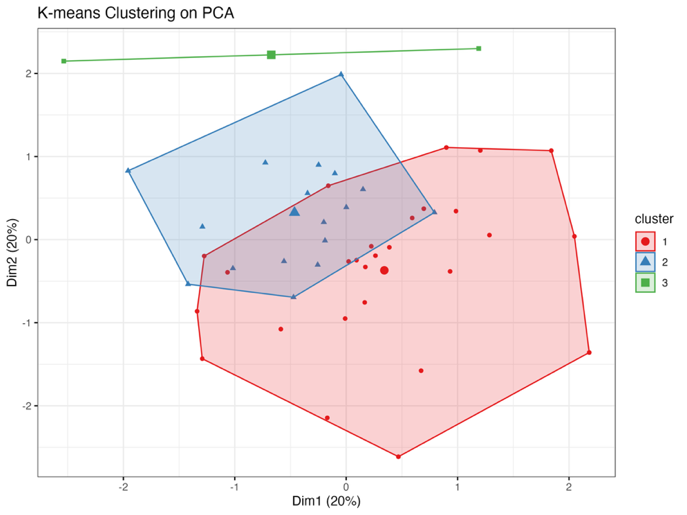

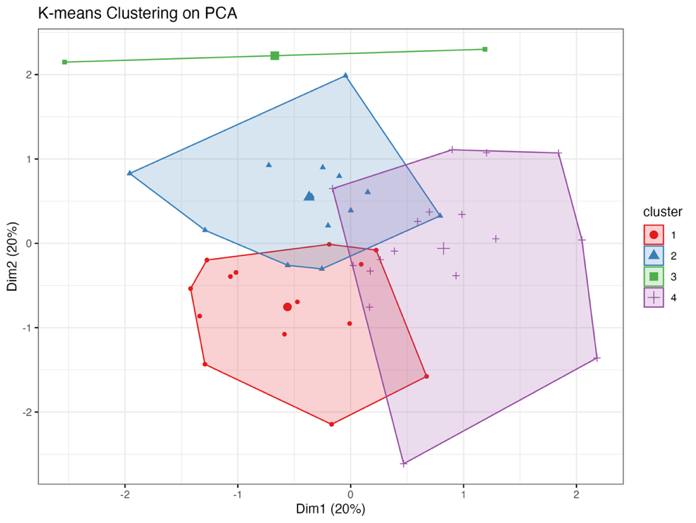

Supplement: S1 Fig — (DOCX) [file pone.0339929.s001.docx]
